# Hyaluronidase and the language of filler reversal: Why “dissolving filler” may be the wrong term

**DOI:** 10.1016/j.jpra.2026.06.013

**Published:** 2026-06-25

**Authors:** Roshan Ravindran, Barbara Pierscionek, Julia Gawronska, Lee Smith

**Affiliations:** a KLNIK, The Colony, Wilmslow, Cheshire, UK; b Faculty of Health, Medicine and Social Care, Anglia Ruskin, Chelmsford, UK; c Centre for Health, Performance and Wellbeing, Anglia Ruskin University, Cambridge, UK; d Department of Public Health, Faculty of Medicine, Biruni University, Istanbul, Turkey

Hyaluronic acid (HA) filler is a widely used tools in aesthetic medicine, and hyaluronidase is the standard intervention when results require correction. The word used almost universally for this process is “dissolve.” Filler in, enzyme in, filler gone. This is the narrative implied by the term “dissolving”, and is often interpreted by patients in precisely these binary terms: filler is either present or absent. “Dissolving” is not an approximate description of what hyaluronidase does. Simplified language plays a legitimate and necessary role in clinical communication, for example, "blood thinner" for anticoagulant is mechanistically defensible. "Dissolving" is not. It does not simplify hydrolysis; it substitutes an entirely different physical process.

Dissolution is a physical process. However, hyaluronidase is an endoglycosidase that cleaves β-1,4 glycosidic bonds maintaining the integrity of the HA polymer chain, fragmenting it into oligosaccharides that are subsequently cleared via lymphatic drainage and receptor-mediated uptake. One process is physical. The other chemical.

The body turns over approximately one third of its HA daily through enzymatic pathways. This process in biochemical literature is consistently termed enzymatic degradation, catabolism, or hydrolysis. The biology has been linguistically precise from the beginning. It was aesthetic medicine that introduced the error, likely when hyaluronidase began to be used off-label for filler correction and “dissolving” was adopted as convenient shorthand. It is worth noting that hyaluronidase has never received FDA approval for the reversal or removal of HA fillers. All use of hyaluronidase for filler management is off-label, and the regulatory language describes the enzyme in terms of absorption and resorption, never dissolution.

The error matters clinically because the word shapes expectation, and expectation shapes consent. Cross-linked HA fillers contain BDDE-mediated bridges that stabilise the polymer into a three-dimensional gel network, physically restricting enzyme penetration and creating wide variability in how readily different products yield to hyaluronidase.

Ultrasound-guided studies confirm that substantial filler remains at twenty-four hours and that complete clearance in a single session is rare.^1^ What clinicians call “dissolving” is staged enzymatic reduction within a complex filler-tissue system. The imaging evidence presented here establishes the biological reality, not the patient interpretation. But it defines the distance between what the word dissolve implies and what the treatment delivers, and it is in that distance that the consent risk sits. The word “dissolve,” taken at face value, implies a completeness that the biology does not reliably deliver, and in many cases multiple treatment sessions are required to approach the result the patient was led to expect from one.

The terminology shapes patient expectation in ways that are difficult to retract once established. It is possible that a patient told their filler will be “dissolved” may expect it to be completely gone. When residual product, oedema, or asymmetry persists, the discrepancy between what was promised and what has occurred undermines trust and complicates the therapeutic relationship. The patient does not think the treatment failed because the enzyme underperformed; they think it failed because the word “dissolve” set an expectation the treatment could not meet. No study has investigated whether the word “dissolving” directly contributes to dissatisfaction or consent disputes in aesthetic practice, and such a study is warranted. However, comprehension of terminology is recognised as one of seven criteria for valid informed consent.^2^

Harris and Weiner’s suggest an alternative, “modifying filler”. This acknowledges that outcomes are not binary.^3^ However, a patient told their filler will be “modified” has no clearer picture of what will happen than before the conversation started.

Not every clinical conversation requires a lesson in polymer chemistry, and there is a legitimate tension between biochemical precision and effective patient communication. But "dissolve" is not inaccurate in a way that only biochemists would notice. It sets a specific expectation, complete removal, that shapes how the patient evaluates the outcome. When medical phrases carry meanings different from their colloquial use, patients frequently misinterpret them, and plainer, more precise phrasing significantly improves comprehension.^4^ Similar patterns have been observed in breast surgery clinics, where only a small proportion of patients correctly understood common clinical terms.^5^ The goal is not to make consent language more technical but to make it true and accessible. “Breaking down the filler” does what clinical language must do. It implies a process rather than an instant event and it does not promise completeness. Indeed, a patient can understand this and importantly they can repeat it accurately to a friend. For professional and regulatory documents, “enzymatic filler degradation” names both the method and the mechanism with the precision those contexts require. Neither term has been formally tested in a patient comprehension study, and such work would help determine which phrasing best supports informed consent.

Aesthetic medicine has matured rapidly, but its terminology has not matured with it. Cardiologists do not call ablation "melting" and Orthopaedic surgeons do not call debridement "cleaning."

We have outgrown "dissolve." The question is whether we replace it with something true or merely something convenient.

## Funding

None.

## Ethical approval

Not required.

## Declaration of competing interest

None declared.

## References

[1] Bravo B.S.F., Cavalcante T., Silveira C. (2024). Resolve and dissolve: an ultrasound-guided investigation on the effects of hyaluronidase on different soft tissue fillers. J Cosmet Dermatol.

[2] Nejadsarvari N., Ebrahimi A. (2014). Different aspects of informed consent in aesthetic surgeries. World J Plastic Surg.

[3] Harris S., Weiner S. (2026). Reframing hyaluronidase in aesthetic medicine: from “dissolving filler” to “modifying filler. Aesthet Surg J.

[4] Gotlieb R., Praska C., Hendrickson M.A. (2022). Accuracy in patient understanding of common medical phrases. JAMA Network Open.

[5] O’Connell R.L., Hartridge-Lambert S.K., Din N., St John E.R., Hitchins C., Johnson T. (2013). Patients’ understanding of medical terminology used in the breast clinic. The Breast.

